# Comparison of the anti-inflammatory and antilipidemic activity of diosmin and saroglitazar in a model of nonalcoholic fatty liver induced by a high-fat diet in Wistar rats

**DOI:** 10.22038/IJBMS.2023.70157.15255

**Published:** 2024

**Authors:** Reza Afarin, Negar Dinarvand, Bahar Jaberian Asl, Ghazal Orak, Elham Shakerian, Fatemeh Bineshfar, Akram Ahangarpour

**Affiliations:** 1 Hyperlipidemia Research Center, Ahvaz Jundishapur University of Medical Sciences, Ahvaz, Iran; 2 Student Research Committee, Ahvaz Jundishapur University of Medical Sciences, Ahvaz, Iran; 3 Diabetes Research Center, Health Research Institute, Ahvaz Jundishapur University of Medical Sciences, Ahvaz, Iran

**Keywords:** Diosmin, Non-alcoholic fatty liver – disease, Non-alcoholic steatohepatitis, Saroglitazar, TGFβ

## Abstract

**Objective(s):**

Non-alcoholic fatty liver disease (NAFLD) is the most common liver-related metabolic disorder in the world, with a global prevalence of 25%. Compounds with anti-inflammatory, lipid-lowering, and insulin-sensitizing properties can be used for the prevention or treatment of NAFLD. Therefore, this study was conducted to investigate the effect of saroglitazar (a dual PPARα/γ agonist) and diosmin (a flavonoid) on non-alcoholic fatty liver induced by a high-fat diet (HFD) in Wistar rats.

**Materials and Methods:**

Forty male Wistar rats (6–8 weeks old) were fed an HFD to induce NAFLD. After 7 weeks, rats were divided into four groups: group1 was fed HFD, and the other groups received HFD+saroglitazar, HFD+diosmin, and HFD+ saroglitazar+diosmin. We examined body and liver weight, histopathology, serum levels of liver enzymes (ALT and AST), and lipid profiles (LDL-C and HDL-C) using the standard protocols. qRT-PCR was also used to examine the expression of PPARα, PPARγ, SREBP1c, FAS, ACC, CPT1α, and pro-inflammatory genes (IL6, TNFα, and TGFβ).

**Results:**

Rats fed the HFD showed characteristics of NAFLD (pathologically and biochemically). Administration of saroglitazar and diosmin alone caused a significant decrease in the levels of PPARγ, SREBP1c, FAS, ACC, ALT, AST, LDL-C, and pro-inflammatory genes and a significant increase in PPARα, CPT1a, and HDL-C in comparison with the HF group (*P*<0.05). Their combined effect was more evident.

**Conclusion:**

Our results showed that diosmin, like saroglitazar, significantly ameliorated inflammatory and lipid profiles in HFD-induced NAFLD, suggesting that diosmin, as a natural compound, could be a suitable alternative to saroglitazar.

## Introduction

Non-alcoholic fatty liver disease (NAFLD) is the most common liver-related metabolic disorder worldwide, with excessive accumulation of fat and no secondary causes (e.g., overconsumption of alcohol) (1, 2), and is characterized by a wide spectrum from mild to severe (non-alcoholic fatty liver (NAFL) to non-alcoholic steatohepatitis (NASH)) (3). NAFLD may lead to cirrhosis and even hepatocellular carcinoma (1, 2). In 2017, NAFLD became a global epidemic and currently has a global prevalence of 25% (2, 3).

Although the molecular mechanisms of NAFLD formation are not known (4), it is mainly associated with many factors, such as inflammation, oxidative stress, insulin resistance (IR), obesity, adipose tissue dysfunction, and endoplasmic reticulum stress (1, 2). Many studies suggest that oxidative stress (OS) caused by reactive oxygen species (ROS) and the resulting inflammation may play a major role in the initiation and development of NAFLD (4). As there is commonly no satisfactory strategy for the treatment of NAFLD, preventing the disease from progressing is essential in the management of NASH (1, 2).

Peroxisome proliferator-activated receptors (PPARs) are transcriptionally regulator of genes related to a wide spectrum of physiological functions including lipid metabolism and inflammation that can be effective in the development of NAFLD and NASH and seem to be attractive targets in their treatment (5, 6). PPAR family exists in three different isotypes: PPARα, PPAR δ/β, and PPARγ, which differ in tissue distribution and function (7, 8). PPARs control physiological conditions by regulating the expression of many genes (9).

PPARα is predominantly present in the liver and muscle, and PPARγ is abundantly present in adipose tissue (6). PPARα is mainly responsible for regulating lipoprotein metabolism (LDL and HDL-C) as well as the rate of fatty acid synthesis and oxidation under various fasting and postprandial conditions to adjust lipid balance (10). PPARα regulates lipogenesis through inhibition of sterol regulatory element binding protein 1 (SREBP1) expression. There are two isoforms of SREBP1: SREBP1a and SREBP1c (11). SREBP-1c in turn controls the expression of fatty acid synthase (FAS) and acetyl coenzyme A carboxylase (ACC) (10, 12), which are the main enzymes in the fatty acid biosynthesis pathway (13). PPARα also regulates fatty acid oxidation by up-regulating the expression of carnitine palmitoyltransferase 1a (CPT-1a), which is involved in the transport of long-chain fatty acids into mitochondria (14). Whereas PPARγ is mainly involved in improving insulin resistance via the regulation of adipokine secretion. Furthermore, both have anti-inflammatory and anti-oxidative properties (14, 11), which act by suppressing the expression of pro-inflammatory genes including IL-1, IL-6, TNF-α, and TGF-β (10, 15, 16).

As a result, it appears that saroglitazar, a dual PPARα/γ agonist, may be useful in the prevention or treatment of NAFLD. On the other hand, the anti-oxidant and anti-inflammatory properties of flavonoids make them effective in inhibiting free radicals and other unstable molecules. Diosmin, as a naturally occurring flavonoid formed in citrus fruits, is currently a natural treatment for many human diseases, including diabetes and chronic venous insufficiency (CVI), and is commonly used for hemorrhoids and leg ulcers that result from poor blood flow. Interestingly, diosmin is also associated with lipid metabolism and plays a role in its improvement (17).

Therefore, considering that the drugs used to treat NAFLD have side effects and no effective treatment has been proposed so far (2), and in order to investigate and compare the biological properties of diosmin and saroglitazar, we studied their effect on non-alcoholic steatohepatitis caused by high-fat diet (HFD) in rats. The HFD contained a high-fat (HF) emulsion that was administered by gavage.

## Materials and Methods

***NAFLD and NASH induction***

A high-fat (HF) emulsion was prepared based on the studies of Zou *et al*. (18) that produces 4243 kcal/L of energy to induce NAFLD and NASH. This emulsion was a mixture of 77% fat, 9% carbohydrates, and 14% protein, supplemented with vitamins and minerals. The emulsion was stored at 4 °C and heated at 42 °C water bath before use.

***Medicines***

Saroglitazar was bought from Cadila Healthcare Limited in Ahmedabad, India. Diosmin was bought from the Iran-based, Abidi Pharmaceutical Company. The medications were dissolved in a 0.5% sodium carboxymethyl cellulose (Na-CMC) solution for injection.

***Animals***

40 adult male Wistar rats (6–8 weeks old, 180–200 g) were obtained from the Experimental Animal Center at Ahvaz Jundishapur University of Medical Sciences. Before starting the study, the rats were adapted to regulated environmental conditions (constant temperature of 25.3 °C, relative humidity of 58%, and a 12-hr light/dark cycle) for one week. The rats were divided into two groups at random: one as a normal control (NC) group (n = 8), fed a standard chow diet, and the other group (n = 32) to induce NASH, fed an HF diet (10 ml.kg^-1^ oral emulsion) and free access to 18% saccharose water for seven weeks. After 7 weeks, two mice from the NC group and four mice from the HF group were sampled to confirm the presence of NAFLD/NASH.

***Treatment***

Rats on the HF diet were randomly assigned to one of four groups. Then, drug treatment was done at the beginning of the eighth week for six weeks by intraperitoneal (IP) injection. Therefore, there were 5 groups, as follows: Group 1 (or NC): received a 0.5% Na-CMC solution. Group 2 (fed HF): received HF diet at 100 mg/kg/day. Group 3 (fed HF): HF plus 3 mg/kg/day saroglitazar. Group 4 (fed HF): HF plus 50 mg/kg/day diosmin. Group 5 (fed HF): HF plus a combination of saroglitazar and diosmin.

***Sample collection***

Rats were euthanized following 6 weeks of drug treatment by an IP injection of a cocktail of ketamine and xylazine at a dose of 90/10 mg.kg^-1^, after 12 hr of fasting. After collecting the blood sample from the aorta, serum separation was performed in a centrifuge at 4500 rpm for 15 min. The removed Liver tissues were washed in an ice-cold saline solution and weighed after drying, and the liver index was determined. After resection, the liver tissue was divided into two parts: one was placed in 10% formalin for histological analysis, and the other was used for RNA extraction and analysis of gene expression.

***Biochemical measurements***

Alanine aminotransferase (ALT) and aspartate aminotransferase (AST) were assessed by a Roche 6000 automated analyzer using respective assay kits. High-density lipoprotein cholesterol (HDL-C) and low-density lipoprotein cholesterol (LDL-C) levels were determined by standard enzymatic colorimetric techniques.

***Gene expression analysis***

Total RNA was extracted from frozen liver tissue using the RNA kit (Yekta Tajhiz, Iran) according to the manufacturer’s instructions. Subsequently, cDNA synthesis from RNA was performed based on reverse transcription using the PrimeScript RT reagent kit (Amplicon, USA). The obtained cDNA was then used to analyze a quantitative real-time polymerase chain reaction (RT-PCR) on an ABI StepOnePlus RT-PCR system. Glyceraldehyde-3-phosphate dehydrogenase (GAPDH) was used as an internal reference to normalize the target mRNA expression. The sequences of the primers utilized (for relative mRNA expression of SREBP-1c, FAS, ACC, PPARγ, PPARα, CPT-1α, IL-1β, IL-6, TNF-α, and TGF-β1) are presented in Table 1. The ΔCt method was used to compare each gene’s expression to that of the internal control gene, GAPDH (ΔCt = Ct gene of target - Ct internal control). The 2^−ΔΔCt^ formula was used to calculate the fold change, which shows the difference in fold expression between the target group and the corresponding control group. Finally, values were reported as mean ± standard deviation (SD).

***Histopathological evaluations***

For histopathological evaluation, liver tissue samples were fixed in 10% buffered formalin. after 24 hr, samples were dehydrated with gradient alcohol and embedded in paraffin wax. Paraffin blocks of liver tissue were cut with a microtome to a thickness of 6-7 μm and placed on a glass slide. Deparaffinization and rehydration with xylene and graded ethanol followed. These slices were stained in two halves with hematoxylin-eosin (HE) and Masson’s trichrome and were histologically examined. Score and grade of the severity of steatosis, inflammation, and fibrosis were reported based on the NASH activity score (NAS) (19, 20).

***Statistical analysis***

The results of different variables were analyzed using t-tests between the NC and HF diet groups, and the comparison of more than two groups was performed using one-way analysis of variance (ANOVA) with the Tukey-Kramer *post hoc* test. Data values were presented as mean ± standard deviation (SD), and *P*-value <0.05 was defined as statistically significant.

## Results

***Changes of body mass and liver index in rats***

**Figure 1:**
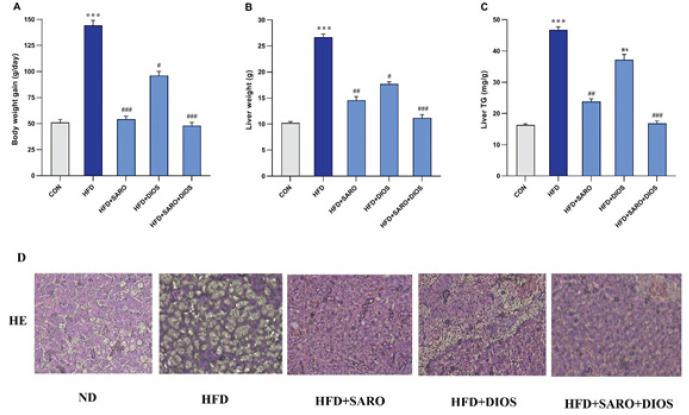
Effects of saroglitazar, diosmin, and combination of diosmin and saroglitazar on body weight (A), liver weight (B), and liver TG (c) in rats after high-fat emulsion and drug administration. (D) Shows hematoxylin–eosin staining of the liver tissue samples

As a result of NASH model induction for 8 weeks, the values of body weight, liver index (liver weight/body weight), and liver TG significantly increased (*P*<0.01) in comparison with the control group. As a result of drug treatment, these variables showed statistically significant changes compared to the HF group (Figure 1).

***Lipid profiles and liver enzymes change after drug treatment***

**Figure 2:**
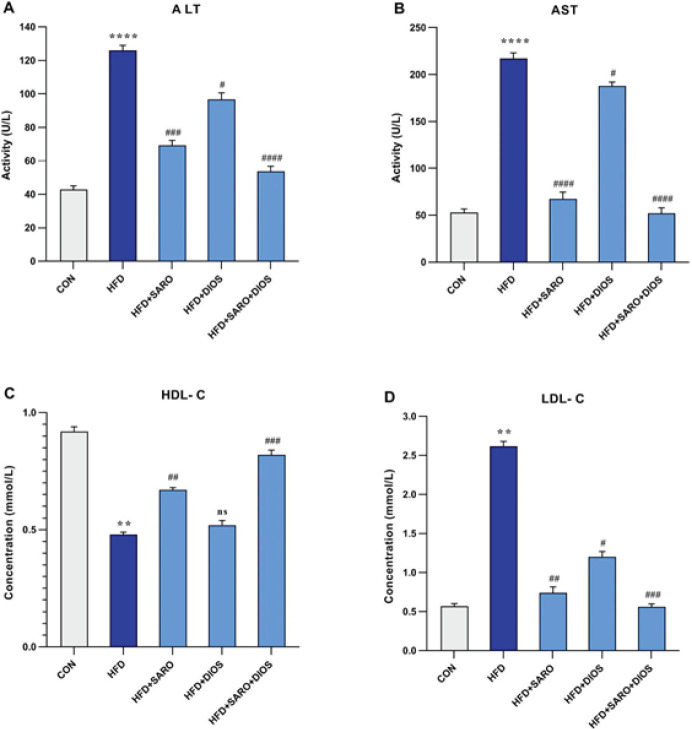
Serum content of ALT, AST, HDL, and LDL levels after high-fat emulsion and drug administration of saroglitazar

Serum AST, ALT, and LDL-C levels in the HF group were significantly higher than in the control group (*P*<0.001, Figure 2 A, B, and D), and HDL-C was significantly lower in the HF group (*P*<0.001, Figure 2 C); while, after treatment with saroglitazar, diosmin, and the combination of saroglitazar and diosmin, levels of AST, ALT, and LDL-C showed a significant decrease compared to the HF group. The increase in HDL-C was also significant in the drug-treated groups. Moreover, these results show that the simultaneous use of saroglitazar and diosmin was more effective than either of them alone.

***Expression of genes involved in lipid metabolism***

**Figure 3:**
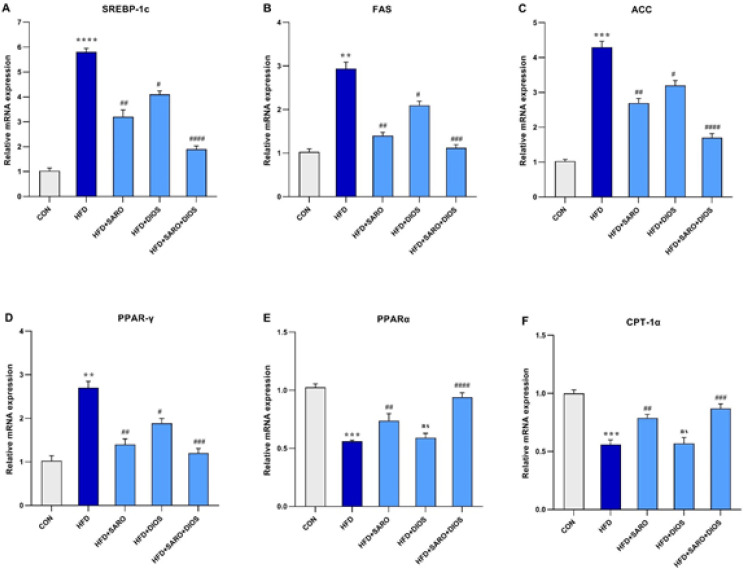
Expression of SREBP-1c, FAS, ACC, PPARγ, PPARα, and CPT-1α in liver tissue after high-fat emulsion and drug administration

The potential effect of HF on lipid metabolism was investigated through the relative expression of the FAS, ACC, CPT1a, SREBP-1c, PPARα, and PPARγ genes in liver tissue specimens. The results show that the expression of the mentioned genes in the HF group was statistically significant in comparison with the normal group (Figure 3; *P*<0.001). On the other hand, treated groups also showed a statistically significant difference in the expression of the mentioned genes in comparison with the HF group. So that the mRNA expression of FAS, ACC, SREBP-1c, and PPARγ genes was significantly higher in comparison with the HF group (*P*<0.05), while the expression of CPT-1a and PPARα genes was significantly lower (*P*<0.05). These changes were clearly observed in the combination of saroglitazar and diosmin.

***Expression of pro-inflammatory genes***

**Figure 4:**
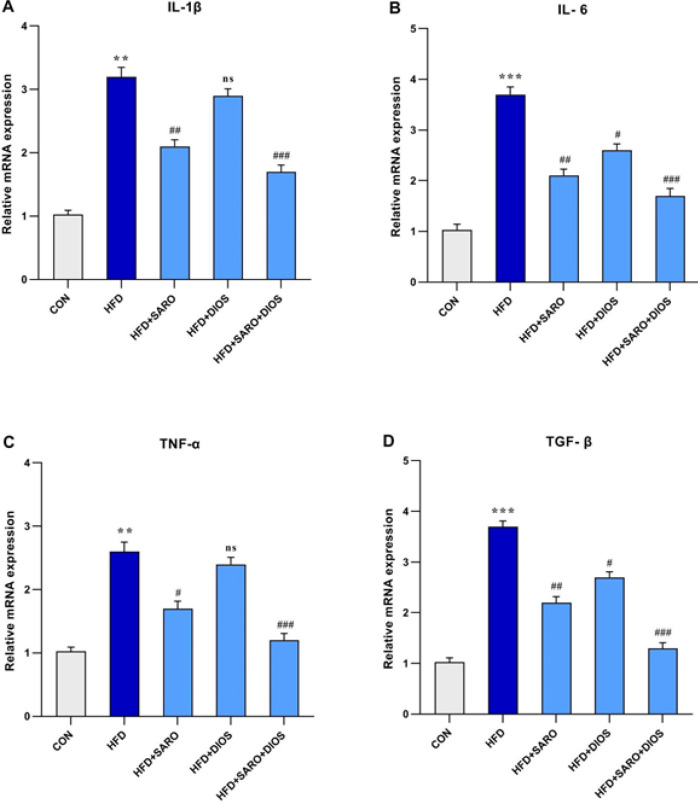
Expression of pro-inflammatory cytokines IL-1, IL-6, TNF-, and TGF-1 in liver tissue following high-fat emulsion and drug administration of saroglitazar

To assess the inflammatory status of rats fed HF, the expression of pro-inflammatory cytokines IL-1, IL-6, TNF-, and TGF-1 was measured (Figure 4), which was significantly higher in comparison with the normal group (*P*<0.05). After treatment, the expression of these genes was significantly decreased in comparison with the HF group (*P*<0.05).

## Discussion

Considering that non-alcoholic fatty liver disease (NAFLD) is the most common liver-related metabolic disorder worldwide and can develop into non-alcoholic steatohepatitis (NASH) (21), and due to the lack of effective treatment (2), it seems necessary to study the therapeutic effects of different compounds. Impaired lipid metabolism and insulin sensitivity are common features in NASH (2), and regulation of the expression of genes involved in these processes is an important function of PPAR𝛼 and PPARγ, respectively (19, 20). In agreement with several previous studies that have demonstrated the role of saroglitazar as dual PPAR-α/γ agonists in regulating the expression of PPARγ and PPARα (22-25), the results of this study also showed that treatment with saroglitazar and diosmin leads to a significant increase and decrease of PPARγ and PPARα expression, respectively, in the HF group. Furthermore, we found a significant decrease in the expression of SREBP-1c, FAS, and ACC and a significant increase in CPT1a after treatment of the HF group with these compounds. As previously mentioned, FAS and ACC are target genes of SREBP-1c and are the main catalysts of the fatty acid biosynthesis pathway (10, 26), whereas CPT-1a catalyzes the initiation step of long-chain fatty acid oxidation (14). We have also shown that saroglitazar and diosmin improved the lipid profile (decrease of LDL-C and increase of HDL-C levels), which confirms the importance of PPARs in lipid metabolism and the improvement of circulating lipoproteins (27). In addition, our results showed a significant decrease in the expression of IL-1β, IL-6, TNF-α, and TGF-β1, supporting the concept that PPARs and flavonoids have anti-inflammatory effects by suppressing a number of inflammatory genes (17, 28). 

Therefore, it is expected that the use of agonists of PPARα and PPARγ is effective in improving NAFLD. In agreement with this hypothesis, we observed significant improvement in histological and pathological features of induced NASH in rats after the administration of saroglitazar. Our finding also showed that diosmin, like saroglitazar, has an effective role in improving NAFLD. 

Consistent with the present findings, Akbari *et al*. have indicated that saroglitazar effectively ameliorates steatosis, fibrosis, and inflammation in HF-induced NASH (21). 

Although several studies and our study have shown that saroglitazar is very effective in reducing inflammation, serum glycemia, lipid parameters, and especially triglycerides and is safe and without any side effects (29), a meta-analysis reported a significant increase in serum creatinine after the use of saroglitazar (30). Other PPAR-α/γ agonists also show renal dysfunction, ALT and AST abnormalities, cardiovascular events, etc. (29). Therefore, according to these findings, it seems that diosmin could be a suitable alternative to saroglitazar and other agonists. 

Our study is corroborated by researchers who have shown that diosmin administration significantly improves liver enzymes and inflammation in iron-induced liver damage (31). Samar H. *et al*. have also shown the beneficial effects of diosmin in the histopathological improvement of NASH (2). Diosmin is a natural flavone glycoside that has anti-inflammatory, anti-diabetic, and anti-oxidant effects, as well as hypolipidemic activities and many other biological activities (32, 33). Our findings confirmed the biological activities of diosmin and also demonstrated its hepatoprotective effect against HF-induced NASH. 

Furthermore, the results of the current study showed that the combination effect of saroglitazar and diosmin is more effective than each one alone. One possible reason is that they act through different mechanisms, although they show the same properties, including improving lipid metabolism and being anti-inflammatory. Therefore, considering that diosmin is a natural and likely safe drug compared to other hepatoprotective drugs, further studies are suggested to understand its mechanisms of action and achieve greater efficacy.

**Table 1:** Primer information for 11 genes

**Gene**	**Forward Primer **	**Reversed Primer**
SREBP-1c	TCTTGACCGACATCGAGACAT	CCTGTGTCTCCTGTCTCACC
FAS	CCCGGACCCAGAATACCAAG	TCTTCAAGTCACACGAGGTG
ACC	TTAAGGGGTGAAGAGGGTGC	CACTTCCAAAGACCTAGCC
PPARγ	CGAGTGTGACGACAAGGTGA	ACGCTTCTTCAATCTGTCTG
PPARα	TGGTGCATTTGGGCGTATCT	CACGAGCGCTAAGCTGTGA
CPT-1α	AGCCCTGAGACAGACTCACA	ATCACGAGGGTCCGTTTTCC
IL-1β	TGCCACCTTTGACAGTGATG	TGATGTGCTGCTGCGAGATT
IL-6	CCAGTTGCCTTCTTGGGACT	TGCCATTGCACAACTCTTTC
TNF-α	ATGGGCTCCCTCTCATCAGT	GCTTGGTGGTTTGCTACGAC
TGF-β1	CTGCTGACCCCCACTGATAC	GGGGCTGATCCCGTTGATT
GAPDH	CTCTCTGCTCCTCCCTGTTC	CGATACGGCCAAATCCGTTC

The amount of each gene was normalized to the amount of Glyceraldehyde 3-phosphate dehydrogenase (GAPDH)

The data values were presented as mean ± SD (n = 8). NC = control group; HFD = high-fat diet; SARO = Saroglitazar; DIOS= diosmin. Significant differences in HFD versus NC (*P*<0.05) are shown with #, and significant differences in HFD versus other groups are shown with *P<0.05; **P<0.01; ***P<0.001.

Means between different groups were analyzed using one-way analysis of variance (ANOVA) with the post hoc Tukey-Kramer test. The data values were presented as the means ± SD. NC = control group; HFD = high-fat diet; SARO = saroglitazar; DIOS= diosmin. Significant differences in HFD versus NC (*P*<0.05) are shown with #, and significant differences in HFD versus other groups are shown with *P<0.05; **P<0.01; ***P<0.001; ****P<0.0001.

The relative expression of genes in mRNA levels was evaluated by real-time PCR, and the results were normalized to the GAPDH signal. The 2-ΔΔCT formula was used to determine the fold change in each group, (mean ± SD). Significant differences in HFD versus NC (*P*<0.05) are shown with #, and significant differences in HFD versus other groups are shown with *P<0.05; **P<0.01; ***P<0.001; ****P<0.0001.

The relative expression of genes in mRNA levels was evaluated by real-time PCR, and the results were normalized to the GAPDH signal. The 2-ΔΔCT formula was used to determine the fold change in each group, (mean ± SD). Significant differences in HFD versus NC (*P*<0.05) are shown with #, and significant differences in HFD versus other groups are shown with *P<0.05; **P<0.01; ***P<0.001.

## Conclusion

Diosmin (a flavonoid), like saroglitazar, significantly ameliorated inflammatory and lipid profiles and showed hepatoprotective effects in NASH. Diosmin also enhanced the effect of saroglitazar more effectively when combined with it. Our findings suggest that the combination of saroglitazar and diosmin may be useful as a therapeutic strategy for NASH, or even diosmin, as a natural compound, could be a suitable alternative to saroglitazar.

## Authors’ Contributions

R A and A A designed the study. R A and N D performed all assays. B JA and GHO analyzed the data. R A wrote the first draft. N D and ESH revised the manuscript. F B contributed to interpreting the results. All authors read and approved the final manuscript.

## Conflicts of Interest

The authors have no conflicting financial interests.
